# A Silicon Valley love triangle: Hiring algorithms, pseudo-science, and the quest for auditability

**DOI:** 10.1016/j.patter.2021.100425

**Published:** 2022-02-11

**Authors:** Mona Sloane, Emanuel Moss, Rumman Chowdhury

**Affiliations:** 1 New York University, New York, NY, USA; 2 Cornell Tech and Data & Society Research Institute, New York, NY, USA; 3 Director of ML Ethics, Transparency and Accountability (META) at Twitter, San Francisco, CA, USA

**Keywords:** audit, accountability, hiring, automated decision-making systems

## Abstract

In this perspective, we develop a matrix for auditing algorithmic decision-making systems (ADSs) used in the hiring domain. The tool is a socio-technical assessment of hiring ADSs that is aimed at surfacing the underlying assumptions that justify the use of an algorithmic tool and the forms of knowledge or insight they purport to produce. These underlying assumptions, it is argued, are crucial for assessing not only whether an ADS works “as intended,” but also whether the intentions with which the tool was designed are well founded. Throughout, we contextualize the use of the matrix within current and proposed regulatory regimes and within emerging hiring practices that incorporate algorithmic technologies. We suggest using the matrix to expose underlying assumptions rooted in pseudo-scientific essentialized understandings of human nature and capability and to critically investigate emerging auditing standards and practices that fail to address these assumptions.

## The bigger picture

Automated hiring tools are increasingly subjected to technical audits for their performance across legally protected groups to ensure that they do not inject additional biases into the hiring process, but these audits often fail to examine the assumptions that underpin the claims made by such tools. This paper outlines a socio-technical approach to audit automated hiring tools. It introduces a matrix that provides a method for inspecting the assumptions that underpin a system and how they are operationalized technically. These assumptions often rest on contradictory or pseudo-scientific theories about job applicants. We offer this matrix to facilitate holistic audits that go beyond technical performance.

## Teaser

This paper outlines a socio-technical approach to holistically audit automated hiring tools. Automated hiring tools are increasingly subjected to technical audits for their performance across legally protected groups to ensure that they do not inject additional biases into the hiring process, but these audits often fail to examine the epistemological assumptions that underpin the claims made by such tools.

## Introduction

Prospective job applicants find their interactions with future workplaces increasingly mediated algorithmically through automated decision-making systems (ADSs) that intervene throughout the hiring process.^1^^,^^2^ These systems have long been used to scan hiring platforms for likely candidates, review resumes to filter in applicants who meet experience and credential criteria, rank applicants, and manage the hiring workflow for hiring managers. More recently, a suite of algorithmic applicant screening technologies is being integrated into the hiring process. These screening technologies variously evaluate applicants by assessing their aptitude for a role through online game playing, analyzing their speech and/or mannerisms to predict on-the-job performance, or by analyzing Meyers-Briggs-styled “personality assessment” questionnaires.^3^ These systems typically use natural language processing (NLP), computer vision, or supervised machine learning techniques that claim to predict job performance based on intonation, written text, micro-expressions, or game performance. Although reliable metrics to evaluate the likely success of applicants remain elusive,^4^ applicant screening and pre-interview evaluation tools are nevertheless being promoted as if they provide stable, reliable, objective, and fair insights into applicants’ suitability for roles without regard for the validity of the evaluative constructs being deployed.^5^^,^^6^ Most worryingly, these constructs are often grounded in pseudo-scientific practices^7^^,^^8^ that recall the dark, eugenicist histories of physiognomy.^9^^,^^10^

Even though there is mounting evidence that such systems harbor bias across demographic categories,^11^ algorithmic and bureaucratic opacity^12^, ^13^, ^14^ have led to slow responses from regulators. Indeed, many of these systems promise more equitable outcomes compared with the purported biases of human decision-makers. Although recent work has examined how hiring managers operationalize concepts like “fairness” through their interactions with these algorithmic systems,^15^ more research is needed to trace the ways claims about the revelatory capacity of hiring ADSs—the claims that they reveal the true potential of job candidates—are shaping the hiring ecosystem. This article contends that audits and assessments of hiring ADSs cannot be limited merely to the degree to which they promote demographic parity within the hiring process but, rather, must also contend with claims that such ADSs can reveal aptitude, future performance, and cultural fit to promote equity and accountability across the entire hiring ecosystem.

## Auditing efforts

In response to investigations that demonstrate a broad range of inequities produced by the integration of ADSs in society,^16^, ^17^, ^18^ a new “algorithmic auditing” industry is blossoming to interrogate and document the potentially discriminatory effects of ADSs.^19^, ^20^, ^21^ Actors within this nascent discipline have been left to their own devices in laying out frameworks and standards to satisfy the demand for audit practitioners coming from industry. This has resulted in what we argue here is an inappropriately constrained form of audit focused on metrics for algorithmic performance.^22^ Brown et al.,^19^ for example, focus on five categories of metrics that robustly characterize the behavior of an algorithmic system, but such metrics are limited in assessing the system within a broader historical or socio-technical context. Algorithmic audits may demonstrate the veracity of a range of claims about an ADS being audited, but their scope is often limited to a narrow range of concerns set in advance by the ADS developer and most often limited to auditing the performance of ADSs with respect to bias across legally protected categories like race and gender (e.g., Wilson et al.^23^).

Recently, there have been significant regulatory proposals that call for algorithmic audits in the United States^24^^,^^25^ and European Union (EU).^26^ Such proposals do not provide clear regulatory definitions of algorithmic audits; proposed bills such as New York City’s bill A1894-2020,^25^ which mandates annual “bias audits” of hiring ADSs, and the proposed Algorithmic Accountability Act in the United States Congress,^24^ which calls for algorithmic impact assessments, do not dictate what precisely they should entail. This is also true of the Regulation on European Approach for Artificial Intelligence, which calls for audits and “conformity assessments” without providing instruction on what these practices ought to entail.^26^ This leaves a regulatory gray area upon which the relevance of such bills hinge. That is, depending on the depth, breadth, and focus of audits as they come into practice through legislation, a bill like A1894-2020 could either enact unprecedented, meaningful steps toward enabling transparency and accountability or create a bureaucratic shield for unscrupulous companies to hide behind by accountability-washing their ADS products. In the latter case, toothless legislation could further deprive affected individuals of their agency within an already opaque, burdensome, and inequitable hiring ecosystem. Developing robust forms of audit and assessment and firmly establishing them within existing industry practices is therefore a crucial step toward ensuring that any forthcoming regulatory and legislative interventions can create meaningful accountability for ADSs.

Currently, there are much-needed ongoing debates about whether algorithmic accountability ought to focus on industry- or sector-specific auditing techniques or on general, all-purpose algorithmic audits.^21^^,^^27^, ^28^, ^29^ Additionally, there are debates about what the proper unit of analysis for algorithmic accountability ought to be. These debates ask whether audits and assessments ought to evaluate a trained algorithmic model, a trained model and the data it was trained on, or a product that may contain multiple interlinked algorithmic models.^19^, ^20^, ^21^^,^^30^ These broader questions about the scope and target of audits are important, but within the hiring industry, there are specific challenges that ought to be considered, and doing so can also shed light on how to address considerations that must be made as part of a regulatory approach to algorithmic accountability.

### Auditing hiring ADSs

Within the hiring domain, companies who use ADSs seldom use one single model for hiring. Instead, they use a suite of ADSs that feed decisions into each other. A job seeker applying for a single open position may encounter different ADSs that recruit them to apply based on their social media profile, that parse their application looking for qualifications and credentials, that subject them to aptitude or personality tests, and that compile a synoptic profile for the hiring manager that will ultimately decide whether to hire them. Therefore, individual ADSs ought to be assessed not as if they are a stand-alone, independent tool but, rather, with consideration of the position they occupy within a linear series of interdependent models that feed one into the other. Put differently, any assessment may treat a tool as its unit of analysis but cannot be assessed as if it operates free from its context of use. In the hiring domain, the outputs of one ADSs are often used as the inputs of another,^31^ and any one of those tools should be evaluated with those dependencies in mind.

From the perspective of job applicants and hiring managers who wish to understand how the tools they use affect their work, the macroscopic process is only as transparent and accountable as its weakest, or most intransparent and unaccountable, ADS. Furthermore, ADS components that are downstream of other components are limited to the outputs of components that are computationally prior to them. Therefore, a tool used in a later stage of hiring may inherit the demographic biases of a tool used at an earlier stage. As an example, assume an organization has a simple hiring pipeline comprised of an ADS that identifies and reaches out to potential candidates encouraging them to apply and an ADS that ranks resumes using natural language processing to evaluate how well that resume fits a job description. If that company audits the resume parsing tool to ensure that it operates in accordance with a corporate value that prioritizes improving gender diversity, that audit would focus on the biases introduced by language models and the potential for gender-based language biases affecting the resume parsing process. However, even if this ADS were assessed and corrected for language biases, it is only as unbiased as the model before it—an unrepresentative sample of the potential applicant pool might have been produced by a biased recruiting tool. If the outreach ADS discriminates against, say, non-male candidates, then the downstream model can only perform to the ceiling set by the prior, biased, model.

Feeding the outputs of the resume parser into a third model (for example, an “emotion detection” ADS that determines candidate trustworthiness by analyzing a video interview), an additional dimension of complexity is introduced in that the “biases” that come into consideration exist not as a function of data or model choice but in the epistemological roots of the system. By “epistemological roots,” we are referring to the claims to knowledge that the system is making—specifically, that there is a way to “know” the interior emotional state of a subject based on externally discernable attributes like facial expressions, pupil dilation, or other physiological characteristics. Such claims have largely failed to demonstrate scientific validity, have not been replicated experimentally,^32^^,^^33^ do not support the additional claims made by vendors that they are useful in predicting on-the-job performance,^34^ and, most troublingly, replicate pseudo-scientific and flawed research that posits imagined links between biology and trustworthiness.^9^^,^^10^ Such a system cannot be assessed as to whether it operates “as intended” if the intention is based on invalid claims about the relationship between appearance and performance. Audits and other investigations that are limited to disparate impact, gender distributions in the data, and the like cannot account for or correct this later tranche of problems.

## The socio-technical matrix for assessing ADSs

Against that backdrop, it is clear that current approaches to audit are inadequate at addressing the entire set of claims made by ADSs if they do not also consider the intentions and claims to knowledge that underlie their operation. If regulation and legislation are to be effective in producing accountability through mandating or recommending audit or assessment, methods for conducting such audits and assessments need to include new ways of framing and understanding how technological systems are encountered in the course of social life. For job applicants’ encounters with technological systems, particularly when those systems build on pseudo-scientific theories, the stakes of inadequate audits and assessments are particularly instructive. To better resolve these stakes, and to aid ongoing efforts to build effective ways of framing and understanding the encounter between job applicants and ADSs, we propose an evaluative matrix for developing a holistic view of hiring ADSs by combining information on its context, its goal, its data, its function, its assumption, and epistemological roots. This matrix can serve as a template for auditors and other assessors but can also be used to support new mechanisms for literacy, accountability, and oversight of ADSs for workers, researchers, policymakers, and practitioners alike. Although other model documentation practices like “datasheets for datasets”^35^ or “model cards for model reporting”^36^ are intended to be compiled by developers, this matrix is intended to also be used by those outside of the development and documentation process to assess already extant algorithmic products.

### How the socio-technical matrix works

The socio-technical matrix is a research tool that can serve as a basis for developing holistic socio-technical assessment and audit methods for hiring ADSs. The matrix can be used by ADS developers, by hiring managers interrogating the ADS tools they use throughout their hiring pipeline, and by the general public (including advocates for job seekers and job seekers themselves). To use the matrix, information on the hiring ADS needs to be collected. In contrast to high-level guidance offered for self-assessment of algorithmic systems,^37^ which ask developers to affirm whether they contemplated various considerations relevant to trustworthy development of algorithmic technologies, the matrix offers prompting questions intended to produce documentary evidence of the answers to these questions and suggests methods for doing so. Access to this information will vary depending on who is using the matrix because companies using such systems are not obliged to disclose to candidates or the public that they are using hiring ADSs or which hiring ADSs they are using. However, information on these hiring ADSs can be found on the Internet as vendors advertise their products and services via case studies or in federal trademark filings.^14^

Some vendors offer a single hiring ADS to be used for a narrow purpose (such as use of a resume parser to narrow down prospects), whereas other companies offer a suite of hiring tools. For the purposes of this paper, the unit of analysis for the matrix is individual ADSs that may be used at specific stages of the hiring process, not the entire process or the company using ADSs (although company-specific uses of the algorithm are relevant for filling out the matrix). The matrix is comprised of seven elements that constitute a description of a hiring system and that need to be assembled to assess its claims.

The matrix prompts an auditor or assessor to compile relevant information about each element and suggests useful questions and methods for obtaining that information (Table 1). The seven elements are as follows:

**Table 1:** Examples of the type of information and ways of obtaining information for each element of the socio-technical matrix

Element	Information	Questions and method
Hiring ADS	name of hiring ADS	question: what is the name of the hiring ADS? method: identify from sales copy
Funnel stage	select from Bogen and Reike^31^	question: at what stage does this company’s hiring ADS operate? method: identify from sales copy and align with funnel list
Goal	narrative description	question: what is the hiring ADS intended to be used for? method: identify from sales copy, interview developers, and hiring managers who operate the hiring ADS
Data	inventory of data types, datasets, and benchmarking datasets	question: what data, and what types of data, are used in training, testing, and operating the hiring ADS? method: interview developers and hiring managers who operate the hiring ADS and inspect data directly
Function	narrative description, machine learning models, and metadata about models	question: how does the hiring ADS work and what is it optimizing for? method: interview developers and hiring managers who operate the hiring ADS and inspect models, metadata, and product directly
Assumption	narrative description	question: why is the hiring ADS useful, what is the assumed relationship between data about an applicant and the goals of the hiring manager, and how does the hiring ADS inform the hiring process? method: interview developers and hiring managers who operate the hiring ADS
Epistemological roots	narrative description	question: where do the assumptions made by the hiring ADS come from, what is their intellectual lineage, and what are the critiques of this lineage? method: archival research, interview developers, and hiring managers who operate the hiring ADS, and ethnographic study of hiring managers and developers

The hiring ADS and funnel stage identify what the hiring ADS is (e.g., Hubert.ai, ZipRecruiter) and how it is intended to be used within the “hiring funnel.” The hiring funnel is a heuristic developed by Bogen and Reike^31^ that segments the hiring process into successive stages in which ADSs are variously employed. These stages are the sourcing of potential job candidates, the screening of candidates to assess their general appropriateness for an open role, the interviewing of applicants to gauge their individual suitability for a position, and the selection of applicants from a small pool of suitable candidates.

The goal of the hiring ADS should clearly state what it aims to do (e.g. “filter the top 1% of applicants while maintaining the diversity of the applicant pool”). These three elements can be derived from sales copy but should be supplemented by interviews with developers and hiring managers who purchase and use the hiring ADS.

Automated hiring ADSs use data. Some hiring ADSs, particularly those that use machine learning or make claims about using artificial intelligence, use data other than that provided by a job applicant to sort, rank, filter, and predict performance for applicants. The matrix should help identify how the data provided by applicants are processed (such as resumes, facial images, voice recordings, chatbot histories, and gameplay).

The function of a hiring ADS is a plain-language description of how it processes data to make its claims (e.g., “compares resumes of previously successful employees to current applicants to predict future success”). Information pertaining to the function of a hiring ADS, how it processes data, and access to the hiring ADS itself should be procured through arrangements with developers and the hiring managers that configure and operate that hiring ADS. The model, or the hiring ADS itself, can subsequently be inspected as a part of an audit or impact assessment by examining the machine learning model that pursues this function in the context of training data, parameter settings, performance characteristics, and its integration into the hiring funnel.

The assumptions that undergird a hiring ADS should capture the logic by which a hiring ADS is seen as useful and can sometimes be derived from sales copy, but a more thorough understanding of a hiring ADS’s assumptions can be gathered from interviewing developers who create a hiring ADS and hiring managers who use a hiring ADS. These assumptions take the form of “this hiring ADS works by comparing the resumes of successful employees to new applicants because successful hires have proven that the attributes documented in their resumes are good predictors of future success.”

Establishing the epistemological roots of a hiring ADS requires archival and/or ethnographic research to outline how a hiring ADS is understood to produce useful knowledge about an applicant. The use of resumes, for example, has a long history in which the resume document itself has been constructed as a reasonable proxy on which to base a hiring manager’s judgements about an applicant that need to be examined in their historical context. Similarly, hiring ADSs that analyze tone of voice to discern personality characteristics have their epistemological roots in psychological profiles of discrete “personality types” and physiognomic approaches that (spuriously) link biological components of facial expression or vocalization to personality.^10^^,^^38^

### Using the matrix

In this section, we demonstrate how the matrix can be used (Table 2). We completed the matrix ourselves, using publicly available information about the product as well as archival and historical research on common claims made by hiring tools. The landscape of automated tools used in the context of hiring is vast and emerging. As discussed above, most companies do not use just one hiring ADS but combine various ADSs at various stages of the talent scouting and hiring process. Therefore, to demonstrate the use of the matrix in this short paper, we focus on “screening,” the second stage of the “hiring funnel.”^31^ This is the stage where candidates are assessed as to whether they are a good match for a job description. This assessment can be based on a myriad of aspects, but in analyzing the narrative description of several ADSs on the market, we identified several goals of available screening ADSs used to predict job fit in hiring: experience, skill, ability, and personality. An individual auditor would discern the specific goal(s) of the ADS as they work through the socio-technical matrix described above.

**Table 2:** An example of a completed socio-technical matrix containing publicly available information for several commercial hiring ADSs

Hiring ADS	Hiretual	Codility	Pymetrics	Humantic
Funnel stage	screening	screening	screening	screening
Goal	experience	skill	ability	personality
Data	resume professional profiles social media profiles proprietary database	coding test exercises	gameplay scores from applicants and workers	resume LinkedIn profile Twitter profile
Function	use profiling for job matching	use test performance for screening candidates in/out	use gameplay performance for screening candidates in/out	use personality profiling for job matching
Assumption	professional and social profile can be matched to job fit	code test performance is a predictor of job skills	gameplay is a predictor of job success	personality is a good predictor for job fit
Epistemological roots	social network theory: the idea that who you are connected with reveals your identity	vocational aptitude testing: the idea that test scores predict ability	eugenics: the idea that intelligence and ability are innate and can be revealed through testing^a^	personality types: the idea that personality is stable over time and a predictor of of performance

a This idea has been well debunked in the social sciences, which posit a critique that rests on such abilities as being socially constructed (Hacking, 1986)^64^.

Experience assessment is the most basic form of assessment used in hiring and often focuses on using an analysis of education, previous positions, and years of experience as a proxy for job fit and future job performance. A standard way in which experience assessments happen is via parsing a resume.

Skill assessment is a form of standardized testing that sets out to measure a candidate’s knowledge and skills that are needed for a particular role. For example, a very common skill assessment for programmers are so-called “coding challenges” where applicants are presented with typical programming challenges and have to solve them live in a job interview.

Ability assessment typically refers to cognitive ability tests. They are different from skill assessments because they do not assess a skill that is learned but are based on the assumption that there are latent mental abilities, such as abstract thinking, attention to detail, aptitude for understanding complex concepts, or adaptability to change, that are not readily discernible from a resume, cover letter, or interview.

Personality assessments set out to determine personality traits in an individual, such as introversion or extroversion. Aside from the infamous Myers-Briggs Type Indicator, a popular taxonomy is the OCEAN model, which models the “big 5 personality traits”: openness to experience, conscientiousness, extraversion, agreeableness, and neuroticism.^39^ In psychology, these personality traits are assumed to be stable,^40^ and in hiring they are therefore thought to be predictive of on-the-job success. Personality tests have a long history in corporate management,^41^ and automating them as part of screening assessments can be seen as falling well into the general shift toward the automation of general managerial decision-making.^42^

To show how the matrix can serve as a way to unpack how ADSs construct experience, skill, ability, and personality, we offer examples from a selection of hiring ADSs marketed by several companies.

## Cues for auditability

The matrix can serve as a tool for developing new avenues for socio-technical work on the auditability of algorithms, beyond the already existing dimensions along which ADSs are currently being audited. It unpacks how the hiring ADS conceptually constructs what it is supposed to measure and rank; for example, experience, skill, ability, and personality.^43^ Social research into the scientific and narrative roots of these frameworks can then help identify *how* theories underlying these measures are translated into constructs that are then operationalized in the hiring ADS.^44^ For example, it allows us to trace how the idea that skill is relevant for assessing job fit became operationalized *as a construct* that underpins standardized assessments, such as standardized tests used in “technical interviews” (see above). This way of mapping the construct genealogies that underlie assessments forms a bridge from qualitative investigation to technical inspection. It focuses attention on interrogating the *construct* as the basis for assessing *validity* without losing sight of the constructs’ epistemological roots.

Validity generally refers to the extent to which a statistical tool measures what it is supposed to measure.^5^ Of particular relevance here is *construct validity*, which is “the extent to which the measure ‘behaves’ in a way consistent with theoretical hypotheses and represents how well scores on the instrument are indicative of the theoretical construct.”^45^

In other words, by using the matrix to clearly articulate what the goals of a particular ADS are *based on the construct that underpins it*, the technical system can be audited for validity (i.e., as to whether it achieves that goal equitably and reliably or even at all). What is important is that the matrix, and the holistic and interdisciplinary approach it promotes, intervenes where a purely technical audit would end up inadvertently promoting a construct that is inherently problematic (e.g. by *exclusively* focusing on assessing construct validity and assessing whether a tool “works as intended” rather than contextualizing the construct with the tool’s intentions and claims to knowledge and their histories). In other words, using the matrix to inform an audit prevents it from taking the construct at face value while still assessing its validity.

Personality testing as it is operationalized within hiring ADSs provides an excellent case study. Here we may use the matrix to identify function, assumption, and epistemological roots to identify the construct that underpins (automated) personality testing: personality is relatively stable; i.e., the idea that personality traits change little over time.^46^ In other words, because some psychology literature assumes that personality is stable over time, although there is mounting disagreement with that,^47^ we can assume that personality *as it is assessed via an ADS* should also be stable across systems, situations, and changing input factors (such as file type). Whether that is the case can be examined in an external audit using the same sample of individuals and their input data (such as CVs) to reveal instability in prediction across trials. Rhea et al. (M.S., unpublished data) have conducted such an audit of a selection of hiring tools and have demonstrated that stability is an important and accessible metric for external auditors seeking to inspect hiring ADS, using these inherent assumptions as baselines for audit design.

The matrix offered here for designing and running socio-technical audits can be used for assessing construct validity of many kinds of aspects that different ADSs measure because constructs carry the implicit claim that they are stable. This is the case for aspects that are measured by assessing candidates but also beyond hiring domains. “Risk” is another such relevant construct that can be made auditable through this matrix, as operationalized in automated risk scoring systems that are used in the criminal justice system,^48^ the public sector,^17^ the healthcare sectors,^49^ or the insurance industry.^50^^,^^51^ For example, *person*-based risk scores that are used in predictive policing^48^ should remain stable if, for example, the ZIP code associated with individual persons changes.

Using the matrix, the audit can then come full circle: audit results can and should be re-contextualized with function, assumption(s), and epistemological roots of that tool to potentially reveal discriminatory effects that the creators of the tool would classify as “unintentional” and that can point toward the construct itself as being problematic or discriminatory by design or, alternately, being sound and necessary for making a hiring decision as required, in the United States by the Civil Rights Act of 1964^52^ and clarified by the Equal Employment Opportunity Commission (https://www.eeoc.gov/prohibited-employment-policiespractices). By helping to identify the constructs that ADSs, and hiring ADSs specifically, claim to measure, the matrix can serve as a basis for more solid validity and reliability assessments that do not end up promoting discriminatory concepts or pseudo-science.

## Toward robust audit frameworks

With the matrix as a starting point, we can better appreciate the complexity of auditing or analyzing hiring ADSs and how this relates to regulatory audit mandates. Although there is no industry-wide agreement on what audits ought to consist of, extant audits may ask about the goal, data, and function of the ADS but generally do not address issues of cross-model contamination (e.g. funnel stage), assumptions, and epistemological roots.

Although the matrix is designed to be used by anyone wishing to conduct an *external* audit, and although audits may be conducted by anyone—developers, companies that purchase ADSs, public advocates, and interested individuals—an ad hoc approach to conducting audits must eventually give way to a consensus about who ought to be tasked with auditing ADSs. Following work on algorithmic impact assessments that calls for multidisciplinary perspectives on identifying algorithmic harms,^53^ regulatory and legislative directives to conduct audits ought to require an interdisciplinary group of experts to conduct audits because of the range of expertise needed to productively interrogate the assumptions on which an ADS is based. In addition to legal and data science teams that are currently engaging in algorithmic audit work, we add our voices to the chorus of calls for addition of social scientists, psychologists, and historians of science and technology to critically evaluate assumptions and epistemologies and inform the audit process as a whole.^30^^,^^53^, ^54^, ^55^, ^56^, ^57^

## Directions for future work

Auditing has also become a bit of a catch-all phrase, and there is value to parsing out different types of audits based on purpose and audience. In fields such as healthcare and finance, where audits are the norm, audit functions can be divided into two audiences: internal and external. Internal auditors are employed by the company, and external auditors can be a regulatory agency or a third-party group. Third-party groups can be a private firm specializing in audits or a potential client that may use the hiring ADS and wants to conduct their own audit. Further, the private firm may be compensated by a potential hiring ADS client or by the company itself.

Whether the audit body is internal or external has significant effects on accessibility to models and data, chronology (i.e., when an audit is conducted in development of this model), the ability to assess cross-model contamination, and incentives.

Internal audit bodies have less external credibility but better access. In general, internal audit bodies serve to ensure that the system is compliant with existing laws and addresses reputational risks. Internal audit groups may have access to data, models, IP, and key employees, which can mean engaging in the earliest stages of development; working closely with developers, data scientists, and project leads at milestones; and mitigating harm before there is adverse impact. These individuals are incentivized to ensure that the company performs well, which can put into question the viability of fundamentally addressing issues of false assumptions and flawed epistemologies. Internal audits can range dramatically because ADSs have no norms or laws dictating audit work. It is rarely the case that internal audits serve as external validation because of conflicts of interest. Generally, internal audits are for organizational purposes; i.e., for product reliability and performance or to ensure that the company avoids legal or reputational backlash when the product is launched.^58^

External audit bodies have more credibility but less access. Even if the audit is paid for by the company, external auditors have pressure to provide quality audit services to retain a good reputation. Regulatory bodies also publish their audit frameworks to allow internal audit teams to ensure adherence, but this also allows public accountability. However, these auditors are not often granted unconstrained access to data, models, IP, or employees, and, in the current regulatory vacuum, companies have a heavy hand in creating these constraints. External audit bodies, however, are better able to critically analyze fundamentals such as assumptions and epistemologies as well as cross-model contamination.^59^ Ultimately, the efficacy of any audit or assessment process will be judged by the material effects the process has on minimization or mitigation of negative effects for society and the environment. The socio-technical matrix proposed here is just that, a proposal, and future work should entail applying the matrix in practice and developing a research program to evaluate its effectiveness at ameliorating harms.

In the matrix, we outline a series of questions that need to be answered to understand how hiring ADSs are intended to work and how they actually operate in practice. Several of those questions require ethnographic investigation into the contextual uses and understanding of these hiring ADSs.

Some aspects of this ethnographic investigation are already well established, particularly for conducting ethnographic interviews,^60^ undertaking workplace ethnography,^61^^,^^62^ and merging ethnographic fieldwork with archival research.^63^ But methodological innovation is called for in tailoring ethnographic interviews, archival research, and fieldwork to robust accountability processes. Additional work is also needed beyond investigation of specific hiring ADSs to better understand how hiring managers, workers, applicants, and others within an organization interact with each other and with hiring tools. Who are the hiring managers using the hiring ADS? When are hiring ADSs used in the pipeline and by whom?

Not all hiring ADSs are used in the same way by hiring managers. Some might take hiring ADSs as suggestions for their own decision-making, others might implement a hiring ADS’s outputs directly, and the ways a hiring ADS functions within a hiring managers workflow ought to be inspected as part of any audit or impact assessment. Building an understanding of how hiring ADSs are used in the workplace is a job for ethnography. Gaining ethnographic insight into how job applicants experience and ascribe meaning to hiring ADS is equally important. Future work can and must focus on this side of the social practice of hiring. This ethnographic insight will be essential not just for designing impactful audits but also for eventually creating less invasive and discriminatory hiring technologies; for example, in collaboration with worker organizations and unions.

## Conclusions

In this paper, we suggest a systematic approach for developing socio-technical assessment for hiring ADSs. We developed a matrix that can serve as a research tool for identifying the concepts that hiring ADSs claim to measure and rank as well as the assumptions rooted in pseudo-scientific essentialized understandings of human nature and capability they may be based on. We argued that the matrix can serve as a basis for more solid validity and reliability assessments as well as a basis for critically investigating emerging auditing standards and practices that currently fail to address issues around pseudo-scientific approaches that essentialize membership in protected categories and make specious inferences from job applicants’ appearances and behaviors.
